# Association between timing of exanthema subitum and febrile seizures: The Japan environment and children’s study

**DOI:** 10.1371/journal.pone.0321061

**Published:** 2025-03-28

**Authors:** Hisao Okabe, Koichi Hashimoto, Mika Yamada, Takashi Ono, Kazufumi Yaginuma, Yohei Kume, Mina Chishiki, Akiko Sato, Yuka Ogata, Karin Imaizumi, Tsuyoshi Murata, Hyo Kyozuka, Yuichi Nagasaka, Seiji Yasumura, Hidekazu Nishigori, Keiya Fujimori, Mitsuaki Hosoya, Hayato Go, for the Japan Environment and Children’s Study (JECS) Group

**Affiliations:** 1 Fukushima Regional Center for the Japan Environment and Children’s Study, Fukushima, Japan; 2 Department of Pediatrics, School of Medicine, Fukushima Medical University, Fukushima, Japan; 3 Department of Obstetrics and Gynecology, School of Medicine, Fukushima Medical University, Fukushima, Japan; 4 Department of Public Health, School of Medicine, Fukushima Medical University, Fukushima, Japan; 5 Fukushima Medical Center for Children and Women, Fukushima Medical University, Fukushima, Japan; Mie University Hospital: Mie Daigaku Igakubu Fuzoku Byoin, JAPAN

## Abstract

Recently, episodes of exanthema subitum (ES) have been occurring later than expected in Japanese patients, though the effects of this delayed timing remain unclear. Therefore, this study examined the association between ES timing and febrile seizure (FS) in Japanese children. Information on the diagnoses of ES and FS was obtained from the Japan Environment and Children’s Study, a nationwide prospective birth cohort study including 97,410 mothers and their children. Two groups were created: early ES (first ES episode between birth and 11 months of age) and late ES (first ES episode between 12 and 23 months of age) groups. Multivariate logistic regression analyses were performed to compare the cumulative incidence rate of FS between the groups. Moreover, because a single episode of fever implies that the ES and FS occurred simultaneously, we examined the association between the ES timing and the simultaneous co-occurrence of FS with ES in a limited cohort with one fever episode until the age of 24 months. In total, 27,238 full-term children were included, with 12,565 (46.1%) in the early ES group and 14,673 (53.9%) in the late ES group. Late ES was estimated to increase the cumulative incidence risk of FS until the age of 24 months (adjusted odds ratio 1.46; 95% confidence interval [1.32–1.61]) and 48 months (1.26 [1.15–1.37]). The limited cohort included 1,022 children, and late ES was estimated to increase the risk of FS with ES (4.39 [1.48–13.02]). Late ES is estimated to increase the risk of FS. Further basic research and cohort studies, including virological examinations, are required to elucidate the pathogenesis of ES with FS.

## Introduction

Febrile seizure (FS) is the most common neurologic disorder in children between the ages of 6 months and 5 years [1], occurring in 2%–4% of children in the West [2–4], 11% of children in Korea [5], and 8%–12% of children in Japan [6,7]. In general, the prognosis for FS is good [1]; however, the burden on emergency medical resources and the psychological burden on the family are high [8]. Of all FS cases, 20%–31% are caused by human herpes virus 6 (HHV-6) infection [9,10].

Exanthema subitum (ES) is an acute febrile disease caused by primary HHV-6 and HHV-7 infections [11–15]; 88%–90% of children develop their first ES episode caused by a primary HHV-6 infection by the age of 2 years [16,17]. Primary HHV-7 infection causes ES or fever without a rash in most children by school age [17–19]. ES can be complicated by FS, which are seizures without identifiable causes such as central nervous system infections or metabolic abnormalities. However, ES can be associated with acute encephalopathy, which can be severe and potentially fatal [20–26].

In recent decades, the first ES episode has been noted to occur at a later age in Japan [27]. The effects of this delayed timing of the first ES episode’s occurrence are unknown, although primary HHV-7 infection is more severe in older children [28]. Therefore, in this study, we examined the association between the timing of the first ES episode and FS in Japanese children in the Japan Environment and Children’s Study (JECS), a nationwide prospective birth cohort study.

## Materials and methods

### Study design and population

Information on ES and FS was obtained through questionnaires used in the JECS, an ongoing nationwide prospective birth cohort study in Japan funded by the Ministry of the Environment, the details of which have been previously described [29–31]. The JECS registered 103,060 pregnancies between January 2011 and March 2014. Information was obtained from pregnant women during their first and second/third trimesters using self-administered questionnaires. Thereafter, detailed information regarding mothers and their children was transcribed from medical records during the first trimester, at delivery, and when the child was 1 month old. After delivery, information was collected at the age of 1 month and every 6 months via self-report questionnaires completed by caregivers.

Statistical analyses were performed using the dataset “jecs-ta-20190930,” which was released in October 2019, and “jecs-qa-20210401,” which was released in April 2021. The datasets contained the data from 104,059 fetuses linked to the respective maternal data collected until the child was 4 years old. Cases of miscarriage, stillbirth, unknown pregnancy outcome, and preterm birth were excluded from the analysis, and children with available data on ES, covariates, and FS were included. Children without an ES diagnosis were excluded because the timing of their ES presentation (early or late) was unclear. Furthermore, we analyzed the association between the timing of ES onset and FS that co-occurred with ES in a limited cohort with one fever episode until the age of 24 months. This is because information on the co-occurrence of ES and FS was unavailable, and a single episode of fever implies that ES and FS occurred simultaneously.

The JECS protocol was reviewed and approved by the Ministry of the Environment’s Institutional Review Board on Epidemiological Studies (No. 100406001) on April 6, 2010, as well as the Ethics Committees of all participating institutions. Research coordinators explained the study to the pregnant women during face-to-face interviews. Written informed consent was obtained from all participants. The JECS was conducted in accordance with the Declaration of Helsinki and other national regulations and guidelines. The authors had no access to information that could identify individual participants during or after data collection.

### Questionnaires and medical records

Information on the presence of older siblings was obtained from the M-T1 questionnaire (completed by pregnant women in their first trimester). Information on the highest level of maternal education, smoking status, and alcohol consumption status was obtained from the M-T2 questionnaire (completed by pregnant women in their second or third trimester). Information on maternal age at delivery, infant’s sex, gestational age, birth weight, and birth season was obtained from medical record transcripts at delivery. Birth seasons were defined as follows: spring (March–May), summer (June–August), autumn (September–November), and winter (December–February). Information on feeding type (breastfeeding, formula milk, or both) was obtained from the medical record transcripts when the child was 1 month old. Information on the ES diagnosis was obtained from the C-6M, C-1Y, C-1hY, and C-2Y questionnaires (completed by caregivers when the child was 6, 12, 18, and 24 months old, respectively). Information on the age at admission to the childcare facility and the incidence of FS was obtained from the C-6M, C-1Y, C-2Y, C-3Y, and C-4Y questionnaires (completed by caregivers when the child was 6, 12, 24, 36, and 48 months old, respectively). The population density of the residences was calculated from the 2010 population and area data obtained from the census for each of the study areas.

### Timing of the first ES episode.

Information on the diagnosis of the ES was collected from the C-6M, C-1Y, C-1hY, and C-2Y questionnaires to determine whether the timing of the first ES episode was between birth and 11 months of age (early ES group) or between 12 and 23 months of age (late ES group). The late ES group included children who did not experience ES during the first 12 months but experienced ES for the first time in their second year of life.

### Outcomes

The incidence of FS at 6, 12, 24, 36, and 48 months was assessed based on a parent-reported doctor’s diagnosis obtained from the C-6M, C-1Y, C-2Y, C-3Y, and C-4Y questionnaires. The primary outcome was the cumulative incidence rate of FS until the age of 48 months, and the secondary outcomes were the cumulative incidence rate of FS until the age of 24 months and the incidence rate of FS between 24 and 48 months of age.

### Statistical analyses

First, the cumulative incidence rate of FS in the early ES and late ES groups was described and compared using the chi-squared test.

Second, the association between the timing of the first ES episode and the cumulative incidence rate of FS until 24 and 48 months of age was examined using multivariate logistic regression models. Based on clinical experience and previous studies [5,32–35], we assessed the following factors as possible covariates in the regression analyses: i) maternal age at delivery; ii) maternal smoking; iii) maternal alcohol consumption; iv) highest level of maternal education; v) population density of residence; vi) sex; vii) birth weight; viii) birth seasons; ix) presence of older siblings; x) feeding type (breastfeeding, formula milk, or both); xi) time of admission to childcare facility; and xii) number of episodes of fever over 38℃ until 24 months of age.

Third, the association between the timing of the first ES episode and the first occurrence of an FS between 24 and 48 months of age was examined using a logistic model.

Fourth, to examine the association between the timing of ES and the co-occurrence of FS with ES, a logistic regression analysis was conducted in the limited cohort with one fever episode until the age of 24 months. The outcome was the cumulative incidence rate of FS until the age of 24 months, which was equated to FS complications associated with ES.

Statistical analyses were performed using the STATA software (version 17.0; StataCorp, College Station, TX, USA). We evaluated the crude odds ratio (cOR) and the adjusted odds ratio (aOR) as measures of association and calculated 95% confidence intervals (CIs).

## Results

### Study participants

**Fig 1:**
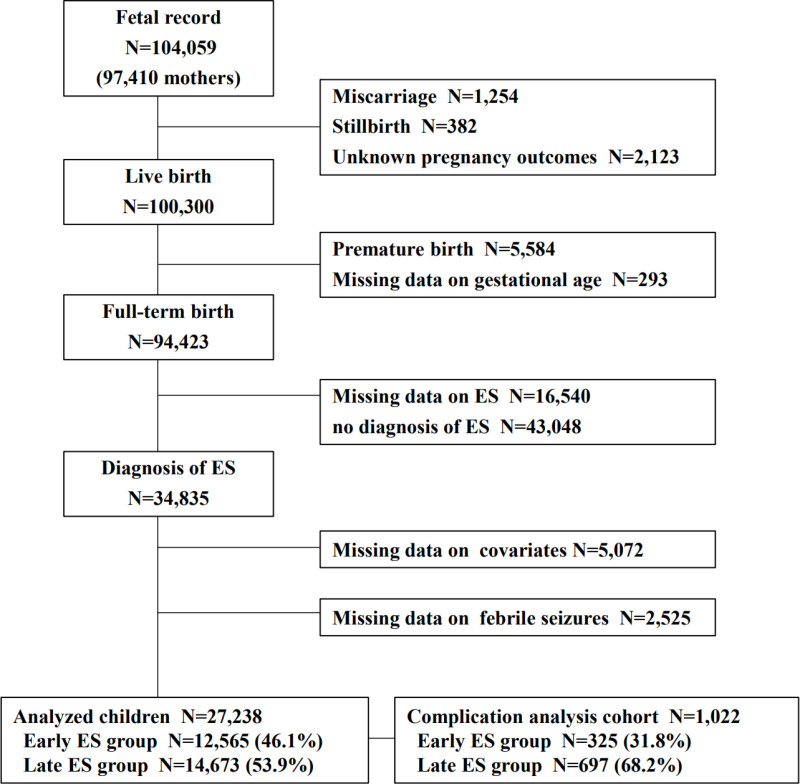
Flowchart of study participant selection. ES, exanthema subitum.

In the current analysis, 27,238 full-term children were included for whom ES, covariate, and FS data were available (Fig 1). The baseline participant characteristics are summarized in Table 1. Among the included participants, 12,565 (46.1%) had their first ES diagnosis from birth to 11 months of age (early ES group), while 14,673 (53.9%) had their first ES diagnosis from 12 to 23 months of age (late ES group).

**Table 1:** Participant characteristics.

	Timing of first ES episode		p-values
	Early ES	Late ES	
**Maternal age (years), mean (SD)**	31.1 (4.7)	31.3 (4.7)	<0.001^§^
**Maternal highest level of education: n (%)**			
**Junior high school**	469 (3.7)	382 (2.6)	<0.001^‡^
**High school**	3748 (29.8)	3965 (27.0)	<0.001^‡^
Technical/vocational college, etc.^*^	5636 (44.9)	6628 (45.2)	0.601^‡^
**Bachelor’s degree or higher**^†^	2712 (21.6)	3698 (25.2)	<0.001^‡^
**Maternal smoking, n (%)**	2033 (16.2)	2108 (14.4)	<0.001^‡^
**Maternal alcohol consumption, n (%)**	6036 (48.0)	7454 (50.8)	<0.001^‡^
**Population density of residence (/km**^**2**^**), mean (SD)**	1,446.6 (2,208.9)	1,435.4 (2,188.5)	0.6747^§^
**Males, n (%)**	6346 (50.5)	7434 (50.7)	0.793^‡^
**Gestational age (weeks), mean (SD)**	39.0 (1.1)	39.0 (1.2)	0.024^§^
**Birth weight (g), mean (SD)**	3,073.9 (367.5)	3,049.0 (365.1)	<0.001^§^
**Birth season, n (%)**			
**Spring (March–May)**	2796 (22.3)	3581 (24.4)	<0.001^‡^
**Summer (June–August)**	3571 (28.4)	3906 (26.6)	0.001^‡^
**Autumn (September–November)**	3493 (27.8)	3937 (26.8)	0.074^‡^
**Winter (December–February)**	2705 (21.5)	3249 (22.1)	<0.221^‡^
**Presence of older sibling, n (%)**	8716 (69.4)	7592 (51.7)	<0.001^‡^
**Methods of feeding, n (%)**			
**Breastfeeding**	7664 (61.0)	8146 (55.5)	<0.001^‡^
**Mixed feeding**	4542 (36.1)	6102 (41.6)	<0.001^‡^
**Infant formula**	359 (2.9)	425 (2.9)	0.847^‡^
**Age at admission to childcare facility, n (%)**			
**No admission until age 2 years**	5060 (40.3)	6383 (43.5)	<0.001^‡^
**From 1.5 to 2 years of age**	1131 (9.0)	1386 (9.4)	0.206^‡^
**From 1 to 1.5 years of age**	1879 (15.0)	2765 (18.8)	<0.001^‡^
**From 6 months to 1 year of age**	3263 (26.0)	3320 (22.6)	<0.001^‡^
**From birth to 6 months of age**	1232 (9.8)	819 (5.6)	<0.001^‡^
**Number of episodes of fever over 38℃ until age 2 years, mean (SD)**	7.0 (5.0)	6.3 (4.8)	<0.001^§^

ES, exanthema subitum, SD, standard deviation.

* Technical junior college, technical/vocational college, and associate’s degree.

† Bachelor’s degree, Graduate degree (Master’s/Doctor’s).

‡ Categorical variables were analyzed using the chi-square test.

§ Continuous variables were analyzed using the t-test.

Compared with the late ES group, the early ES group had younger and less educated mothers, higher rates of maternal smoking, lower rates of maternal alcohol consumption, greater birth weights, more summer-born infants, more older siblings, higher rates of breastfeeding, more frequent fevers, and earlier admission to childcare facilities (Table 1).

### Association between the timing of the first ES episode and incidence rate of FS

**Fig 2:**
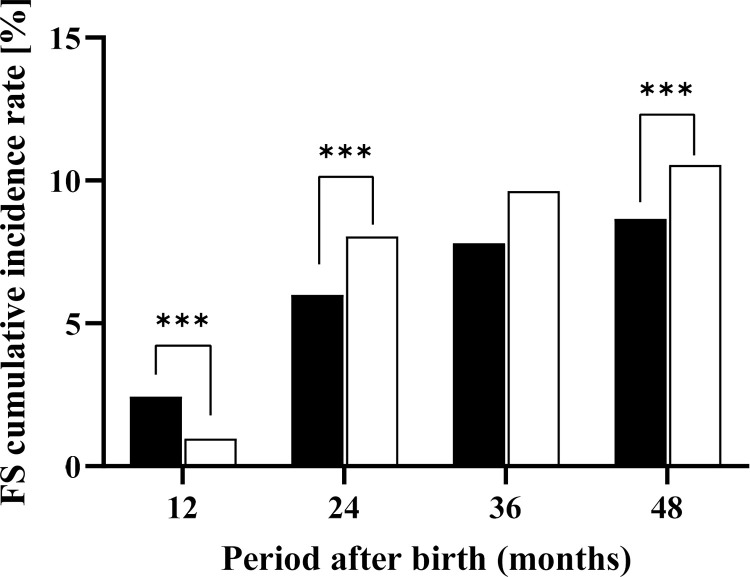
Trends in the cumulative incidence rate of febrile seizure based on the timing of the first exanthema subitum episode. ES, exanthema subitum. *** p <  0.001. The black bars indicate the early ES group (first ES episode occurring between birth and 11 months of age), while the white bars represent the late ES group (first ES episode occurring between 12 and 23 months of age).

The cumulative incidence rate of FS at 12 months was higher in the early ES group than in the late ES group (2.4% vs. 1.0%, p < 0.001). However, after 24 months, the cumulative incidence rate was higher in the late ES group than in the early ES group (early ES group vs. late ES group at 24 months, 6.0% vs. 8.0%, p < 0.001; and at 48 months, 8.7% vs. 10.5%, p < 0.001) (Fig 2). We described the cumulative incidence rate of FS for a cohort of 94,423 children with full-term birth before excluding cases with missing data. The results revealed an incidence of 1.2% at 12 months, 5.6% at 24 months, 7.5% at 36 months, and 7.7% at 48 months. These findings indicated that the proportion of children developing FS was particularly high between 12 and 23 months of age (S1 Fig).

Using logistic regression models, late ES episodes were estimated to increase the cumulative incidence risk of FS until the age of 24 and 48 months (until age 24 months, aOR: 1.46, 95% CI: [1.32–1.61]; until age 48 months, 1.26 [1.15–1.37]) (Table 2).

**Table 2:** Association between the timing of the first ES episode and the incidence rate of FS.

Timing of first ES	Case, n	Subtotal, n	Incidence rate, %	cOR (95% CI)	aOR (95%CI)	p-values
**Cumulative incidence rate of FS from birth to 24 months of age**						
**Early ES**	753	12,565	6.0	Reference	Reference	<0.001
**Late ES**	1180	14,673	8.0	1.37 (1.24–1.50)	1.46 (1.32–1.61)	
**Cumulative incidence rate of FS from birth to 48 months of age**						
**Early ES**	1087	12,565	8.7	Reference	Reference	<0.001
**Late ES**	1547	14,673	10.5	1.24 (1.14–1.35)	1.26 (1.15–1.37)	
**Incidence rate of FS between 24 and 48 months of age**						
**Early ES**	334	12,062	2.8	Reference	Reference	0.305
**Late ES**	367	13,711	2.7	0.96 (0.82–1.11)	0.92 (0.79–1.07)	

aOR, adjusted odds ratio, CI, confidence interval, cOR, crude odds ratio, ES, exanthema subitum, FS, febrile seizure.

The FS incidence rate between 24 and 48 months was 2.8% and 2.7% in the early and late ES groups, respectively. In logistic regression analysis, no significant association was observed between the timing of the first ES episode and the FS incidence rate between ages of 24 and 48 months (0.92 [0.79–1.07]) (Table 2).

The association between the timing of the first ES episode and the cumulative incidence of FS was examined using multivariate logistic regression models, with adjustment for the following factors as possible covariates: i) maternal age at delivery; ii) maternal smoking; iii) maternal alcohol consumption; iv) highest level of maternal education; v) population density of residence; vi) sex; vii) birth weight; viii) birth seasons; ix) presence of older siblings; x) feeding type (breastfeeding, formula milk, or both); xi) time at admission to childcare facility; and xii) number of episodes of fever over 38℃ until 24 months of age.

### Association between the ES timing and FS that co-occurred with ES

In the analysis of the limited cohort of 1,022 children, 35 (3.4%) had FS that co-occurred with ES. The FS co-occurrence rate was higher in the late ES group (4.4%) than in the early ES group (1.5%). Using logistic regression models, late ES episodes were estimated to increase the risk of FS co-occurrence (aOR: 4.39, 95% CI [1.48–13.02]) (Table 3).

**Table 3:** Association between the timing of exanthema subitum and FS co-occurrence from birth to 24 months of age.

Timing of first ES	Case, n	Subtotal, n	FS co-occurrence rate, %	cOR (95% CI)	aOR (95% CI)	p-value
**Early ES**	4	325	1.2	Reference	Reference	0.008
**Late ES**	31	697	4.4	3.73 (1.30–10.67)	4.39 (1.48–13.02)	

aOR, adjusted odds ratio, CI, confidence interval, cOR, crude odds ratio, ES, exanthema subitum, FS, febrile seizure..

The association between the timing of the first ES episode and the cumulative co-occurrence rate of FS was examined using multivariate logistic regression models, with adjustment for the following factors as possible covariates: i) maternal age at delivery; ii) maternal smoking; iii) maternal alcohol consumption; iv) highest level of maternal education; v) population density of residence; vi) sex; vii) birth weight; viii) birth seasons; ix) presence of older siblings; x) feeding type (breastfeeding, formula milk, or both); xi) and time at admission to childcare facility.

## Discussion

The association between the timing of the first ES episode and FS in Japanese children was examined using data from the JECS. The cumulative incidence risk of FS until 24 and 48 months of age was higher for children with late ES episodes than for those with early ES episodes; the same trend was observed for FS that co-occurred with ES. To the best of our knowledge, this is the first study worldwide to examine the association between the timing of the first ES episode and FS and is expected to pave the way for further research.

Hall et al. reported FS in 10% of children with HHV-6 infection who presented to the emergency department [9]; this proportion was higher than the 3.4% observed in the FS complication analysis in this study. This difference may include contributions from limitations related to children who visited the emergency room and differences in the study populations; the present study only included children who experienced a single febrile episode by 24 months of age. Although Hall et al. did not report an association between the timing of HHV-6 infection and FS complications, this study suggests that ES after 12 months of age is a risk factor for FS complications. Suga et al. reported that 21 of 105 infants with FS had a primary HHV-6 infection. Of the 21 participants in their study with FS and primary HHV-6 infection, 13 were younger than 12 months and eight were 1–2 years old [10]. Because the number of participants with primary HHV-6 infection without FS was unclear, the FS co-occurrence rate could not be calculated. However, according to the pediatric sentinel points from 2000 to 2009 in Japan, the number of children under 12 months of age with ES was twice that of children 1–2 years of age [36], suggesting a high co-occurrence rate of FS with ES in 1–2-year-olds, which supports the results of this study.

The mechanism through which the timing of the first ES episode is associated with the cumulative incidence rate of FS remains unclear. Our analysis showed that the co-occurrence rate of FS with ES was higher in the late ES group than in the early ES group. In contrast, no association was observed between the timing of the first ES episode and the FS incidence rate between 24 and 48 months of age (not FS that co-occurred with ES). These results suggest that FS that co-occurred with ES, but not FS without ES, is associated with ES episode timing. Several hypotheses have been proposed to explain the increase in the incidence rate of FS that co-occurred with ES in participants with late ES. The first hypothesis suggests that the risk of FS complications is greater if ES occurred during periods when FS tended to occur. FS most likely occurs at 12–23 months of age [5,10]. FS, with a peak incidence between 12 and 18 months of age, likely results from the vulnerability of the developing central nervous system to the effects of fever, combined with an underlying genetic predisposition and environmental factors [37]. Therefore, it is likely that 12–23-month-old children were prone to FS caused by high fever attributed to ES. The second hypothesis was that late ES tends to lead to neurological complications. HHV-7 infection, one of the causes of ES, is more severe in older children [28]. Data on ES after the age of 2 years were unavailable in this study; therefore, whether the risk of FS increases even at older ages is unclear.

The strengths of this study include the use of a large sample of the general population across Japan and the inclusion of various factors. Several potential confounding factors could influence the association between ES and FS, making it essential to adjust for these confounders in the analysis. The widest range of information covered potentially confounding information, allowing for adjustment for the covariates. Moreover, the study design involved questionnaire responses obtained every 6 months; this reduced recall bias as much as possible. While a 6-month interval may not completely eliminate recall bias, it strikes a balance between minimizing participant burden and collecting data at reasonable intervals to ensure the feasibility of a large-scale study.

This study examined, for the first time to our knowledge, the association between the incidence rate of FS and the timing of ES, which was the main cause of FS, and showed that late ES might increase the risk of FS. In Japan, the incidence rate of late ES has increased in recent decades [27]; the prevalence of FS was 8.3% in the 1970s and 12.3% in 2016–2017 [6,7], suggesting an increase. Our findings suggested that late ES is associated with an increased prevalence of FS. The results of this study will provide a foundation for further research aimed at reducing the burden of FS and ES. While most ES cases have a good prognosis, in rare cases, severe neurological complications can cause death or neurological sequelae [20–26]. Elucidation of the mechanisms underlying these severe neurological complications is expected to help establish methods for the prevention and treatment of severe ES. The new finding that late ES might increase the risk of FS complications is expected to serve as a baseline for further studies to elucidate the underlying mechanisms.

However, this study had some limitations. First, information on ES and FS diagnoses was based on a questionnaire survey without virological examination. Because the phenotype of primary HHV-6 infection may vary with age [27], we cannot exclude the possibility that the results will differ in studies employing virological examinations. In addition, it is unclear whether the diagnosis of FS adhered to the criteria in clinical guidelines. Second, information on whether FS was caused by ES was lacking, and only a limited cohort with one fever episode until the age of 24 months could be examined for FS that co-occurred with ES. This limitation in the cohort may have introduced selection bias, potentially affecting the generalizability of the findings. Third, because data on ES after 24 months of age were lacking, whether ES is more likely to be complicated by FS in children older than 24 months could not be examined.

## Conclusion

In the present study the incidence risk of FS until 24 and 48 months of age was higher for children in late ES group than for those in early ES group; the same was noted for FS that co-occurred with ES.

Further studies, such as cohort studies on the neurological complications of ES, including virological examinations, and basic research, including animal studies, are needed to elucidate the pathogenesis of ES and FS.

## Supporting information

S1 Fig Cumulative incidence rate of FS.
FS, febrile seizure. We described the cumulative incidence rate of FS among a cohort of 94,423 full-term birth children before excluding cases with missing data. The results revealed an incidence of 1.2% at 12 months, 5.6% at 24 months, 7.5% at 36 months, and 7.7% at 48 months.
(TIF)
