# Supplementary figures and images for: Association between timing of exanthema subitum and febrile seizures: The Japan environment and children’s study
Source: PLoS One. 2025 Mar 28;20(3):e0321061. doi: 10.1371/journal.pone.0321061 (PMC11952210; doi:10.1371/journal.pone.0321061)

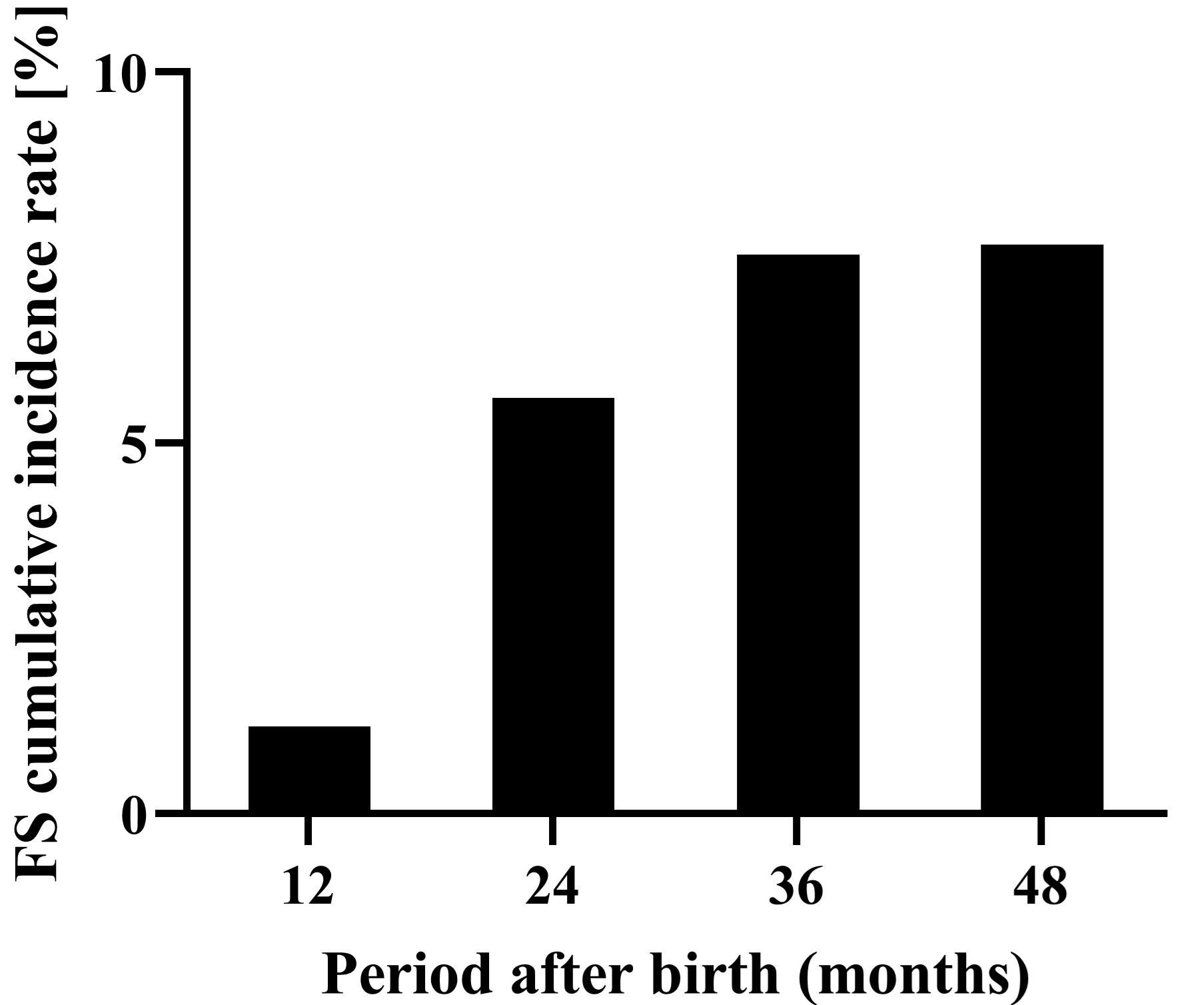

Supplement: S1 Fig — FS, febrile seizure. We described the cumulative incidence rate of FS among a cohort of 94,423 full-term birth children before excluding cases with missing data. The results revealed an incidence of 1.2% at 12 months, 5.6% at 24 months, 7.5% at 36 months, and 7.7% at 48 months. (TIF) [file pone.0321061.s001.tif]
